# Supplementary material for: Structural and biochemical insights into lipid transport by VPS13 proteins
Source: J Cell Biol. 2022 Mar 31;221(5):e202202030. doi: 10.1083/jcb.202202030 (PMC8978259; doi:10.1083/jcb.202202030)
Supplement: Table S1 — lists primers for the study [file JCB_202202030_TableS1.docx]

Supplementary Table 1. Primers for the study.

| VABct | FP | actttaagaaggagatataccatggtagcgccatacaggatcagg |
| --- | --- | --- |
|  | RP | gttcgacttaagcattatgcggccgcttattagtgatggtgatggtgatgctggcggtacaggctctttg |
| PXP(Mcp1ct)_VABct | FP | catcgccgaggatgccgtcgtcgacgcgccatacaggatcagg |
|  | RP | ggggcaggatccaactcgaccagcgataccatggtatatctccttcttaaag |
| VABct_PxP(Mcp1ct) | FP | gtcgagttggatcctgcccccatccatcaccatcaccatcactaataag |
|  | RP | cagcgagacggccgagacgagcgtctggcggtacaggctctt |
| VABct_R2489E | FP | gaacagcggctgatccgcgtggag |
|  | RP | ctgcccagcgcgagcaatttttag |
| VABct_mutLIM | FP | ccgcgtagtgcagcgccgtatgcctgggacttcccggctgcgaag |
|  | RP | gggcgcacggtaccggacaggacgccaaccggagcggtc |
| VABsc | FP | tttaagaaggagatatacatatgaagccatatcaactggtaaac |
|  | RP | tggtggtggtggtgctcgagattggccttatagttaacaataactaag |
| PxP(Mcp1sc)-VABsc | FP | agtagaccctgctagtctccctcatgataagccatatcaactggtaaac |
|  | RP | ggttctggaggcacttcatgcaactttatcatatgtatatctccttcttaaag |
| PxP(Ypt35sc)-VABsc | FP | catccaactacttgacgaagactccacggagaagccatatcaactggtaaac |
|  | RP | ggttcgggaggtagaaaggatatcttgtcgctcatatgtatatctccttcttaaag |
| PxP(Spo71sc)-VABsc | FP | atggcctaccgaactgacggaggaagagagaaagccatatcaactggtaaac |
|  | RP | ggaggtaggatattaataaacggaagatgactcatatgtatatctccttcttaaag |
| VABsc_R2396E | FP | gaacataagcttttaaagattgaaattcttttggacaaagc |
|  | RP | tgaatttttcaaaactttcaaatatgttacaccgacatttttg |
| VABsc_mutIIM | FP | tcgaaaagtgcggcgccatacgcatgggattttcctacagctaaggag |
|  | RP | gggcgccctgtaaaagataggtttgaaactacggcttga |
| Mcp1sc | FP | aggggccccttgcggccgccatgataaagttgcatgaagtgcc |
|  | RP | agggatgccacccgggatccctaattcacgtgcaacagc |
| XK | FP | ccaggggccccttgcggccgcgatgaaattcccggcctcg |
|  | RP | agggatgccacccgggatccttaagcagagcagagatcttc |
