# Supplementary material for: Structural and biochemical insights into lipid transport by VPS13 proteins
Source: J Cell Biol. 2022 Mar 31;221(5):e202202030. doi: 10.1083/jcb.202202030 (PMC8978259; doi:10.1083/jcb.202202030)
Supplement: Table S2 — lists data collection and refinement statistics [file JCB_202202030_TableS2.docx]

Supplementary Table 2: Data collection and refinement statistics.

| **Crystal parameters** |  |
| --- | --- |
| Space group | P 1 21 1 |
| Cell dimensions |  |
| a, b, c (Å) | 83.735, 91.225, 132.769 |
| α, β, γ (°) | 90.000, 102.163, 90.000 |
| Monomers/ASU | 2 |
| **Data collection** |  |
| Wavelength (Å) | 0.979180 |
| Resolution range (Å) | 48.38-3.00 (3.11-3.00) |
| Completeness (%) | 98.5 (97.6) |
| Redundancy | 3.5 (3.6) |
| I/σ(I) | 17.2 (3.4) |
| Rmerge (%) | 4.4 (32.2) |
| Rmeas (%) | 5.2 (37.8) |
| Rpim (%) | 2.8 (19.7) |
| CC (1/2) | 0.999 (0.970) |
| Refinement |  |
| Resolution range (Å) | 48.36-3.00 (3.07-3.00) |
| Rwork (%) | 24.96 |
| Rfree (%) | 28.48 |
| RMSD bond angles/lengths | 0.852/0.005 |
| Ramachandran statistics (% in favored  /allowed/other regions) | 95.96/3.86/0.18 |
| PDB accession code | 7U8T |
