# Supplementary material for: Structural and biochemical insights into lipid transport by VPS13 proteins
Source: J Cell Biol. 2022 Mar 31;221(5):e202202030. doi: 10.1083/jcb.202202030 (PMC8978259; doi:10.1083/jcb.202202030)
Supplement: SourceData F3 — contains original blots for Fig. 3. [file JCB_202202030_SourceDataF3.pdf]

SourceData for Figure 3A

Mcp1:

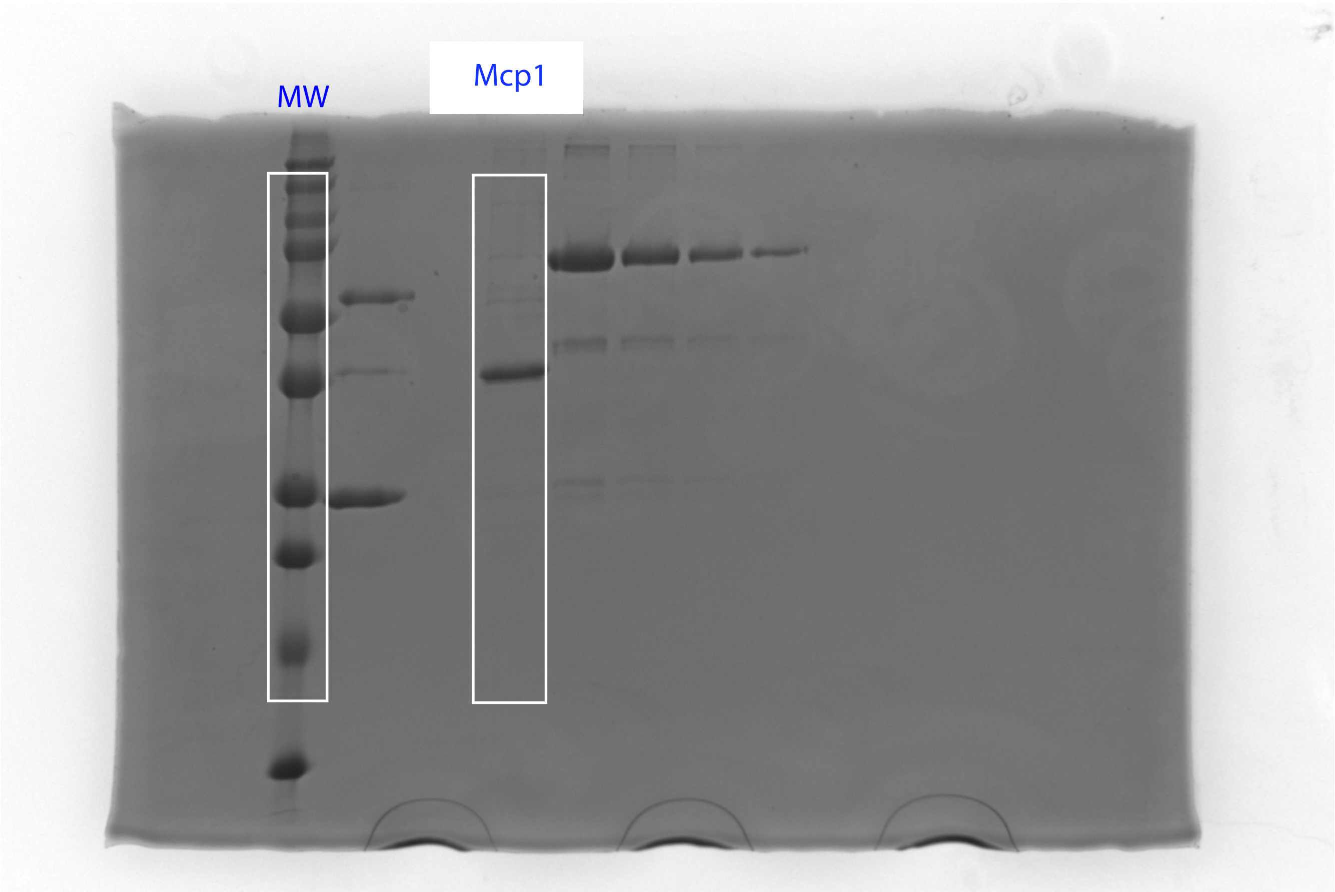

Coomassie stain

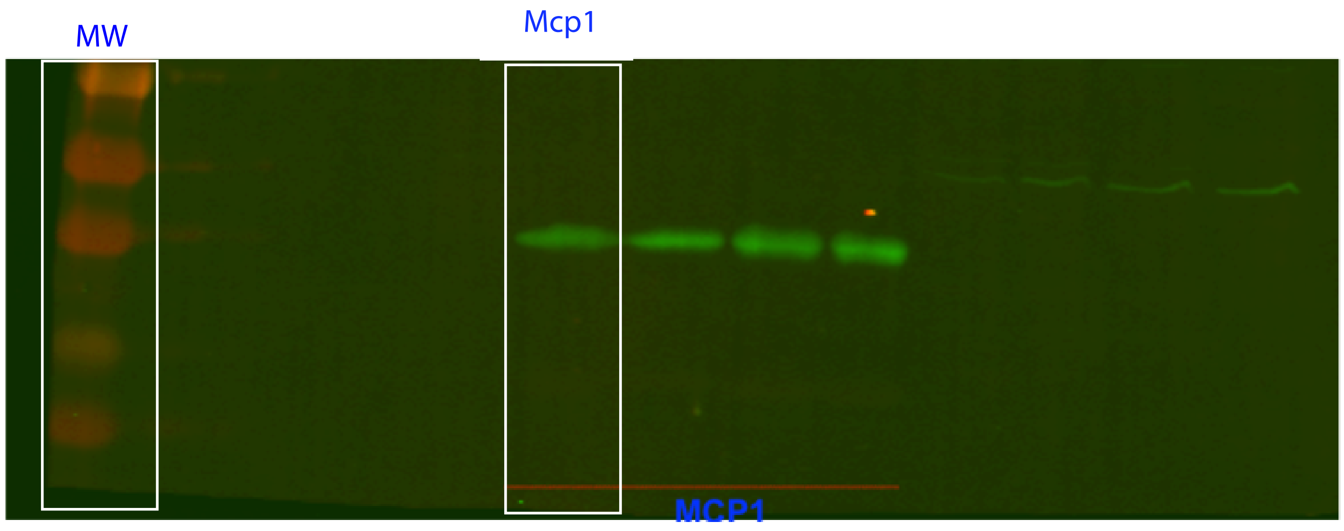

Anti-FLAG Western

XK:

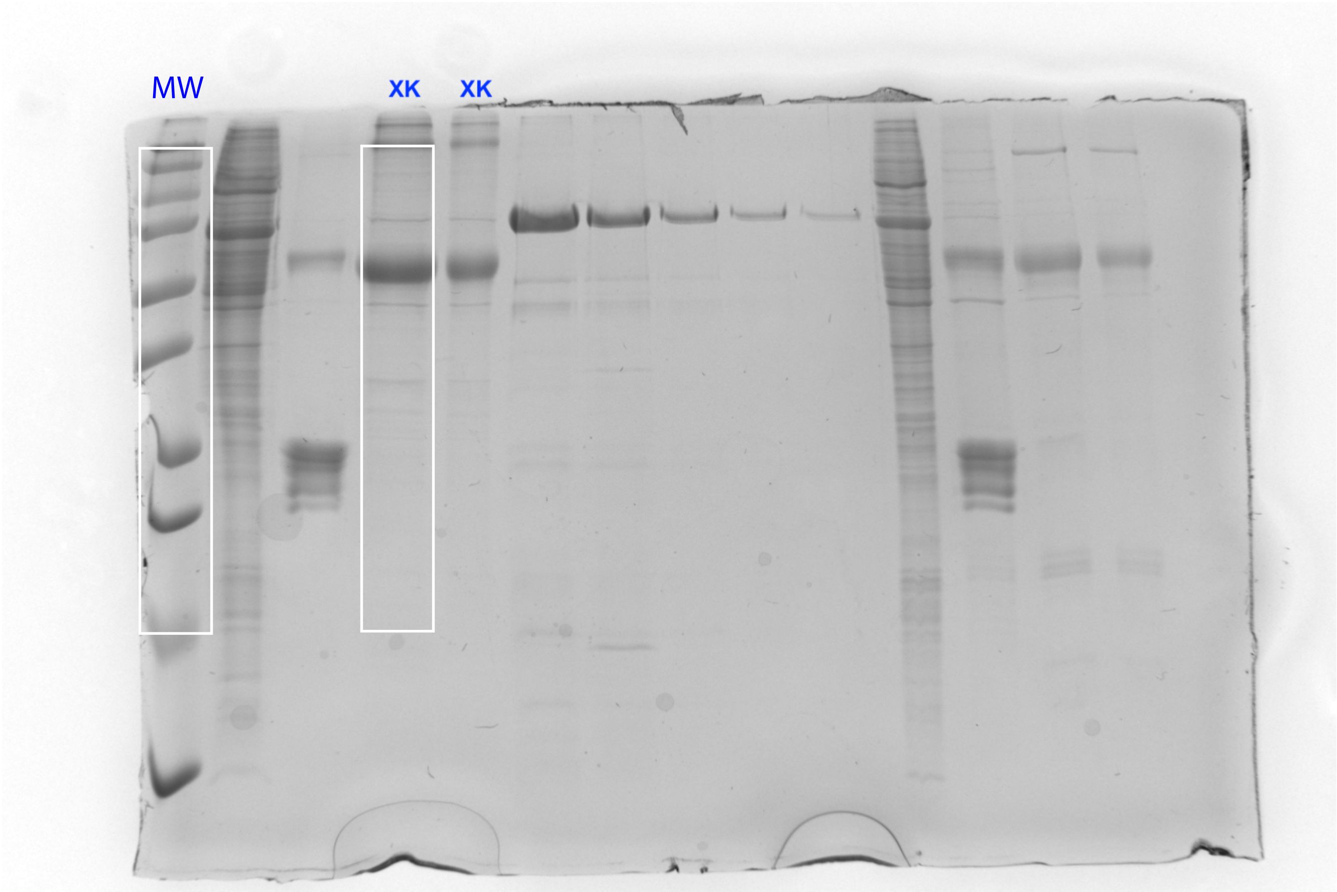

Coomassie stain

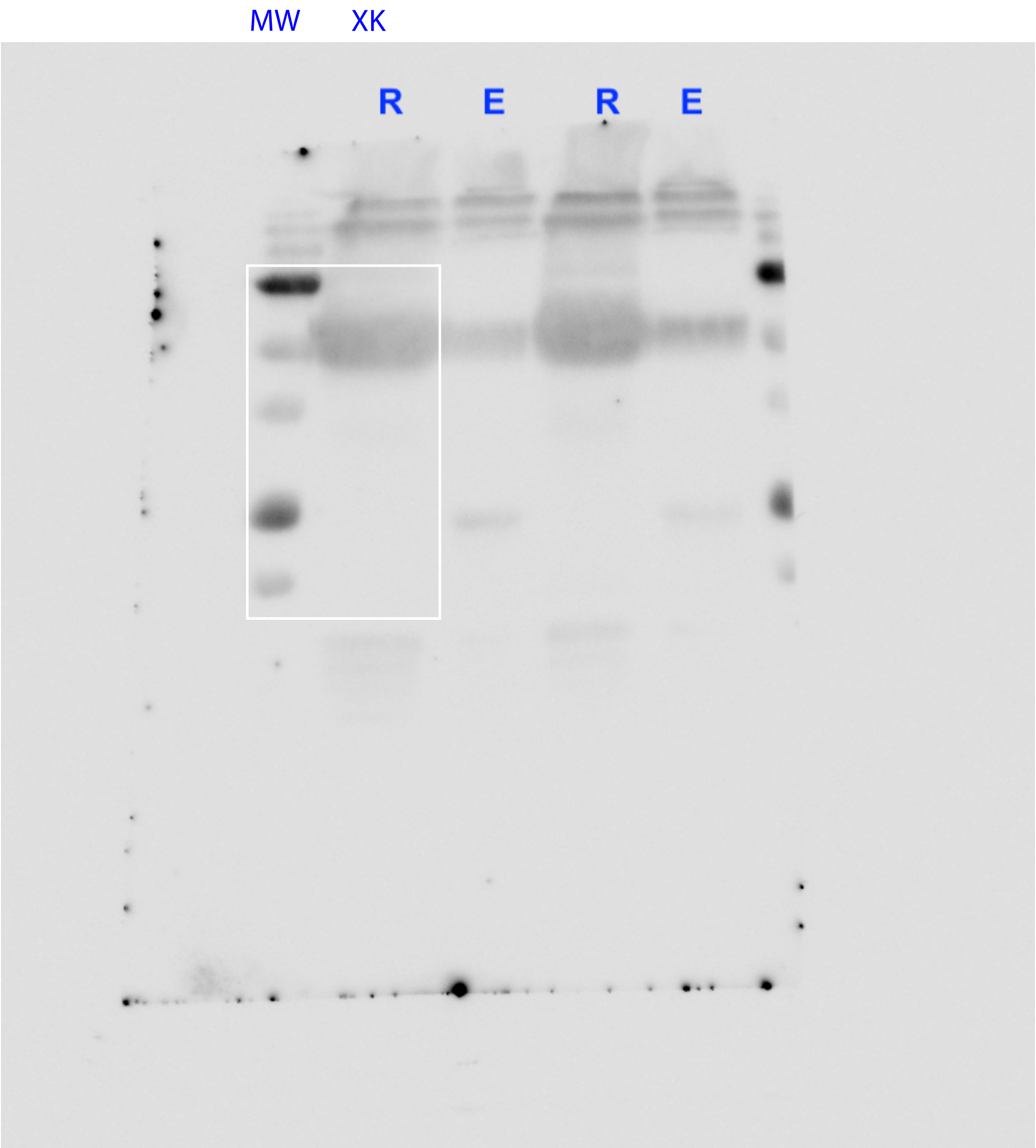

Anti-FLAG Western
